# A prospective, multicenter, single-arm clinical trial cohort to evaluate the safety and effectiveness of a novel stent graft system (WeFlow-JAAA) for the treatment of juxtarenal abdominal aortic aneurysm: A study protocol

**DOI:** 10.3389/fcvm.2022.1013834

**Published:** 2022-09-28

**Authors:** Jiang-Ping Gao, Hong-Peng Zhang, Xin Jia, Jiang Xiong, Xiao-Hui Ma, Li-Jun Wang, Min-Hong Zhang, Yong-Le Xu, Wei Guo

**Affiliations:** ^1^Department of Vascular Surgery, Chinese PLA General Hospital, Beijing, China; ^2^Medical School of Chinese PLA, Beijing, China

**Keywords:** abdominal aortic aneurysm, juxtarenal, endovascular aortic repair, endovascular repair, WeFlow-JAAA

## Abstract

**Introduction:**

Juxtarenal abdominal aortic aneurysms (JRAAAs) are challenging to cure by traditional endovascular aortic repair (EVAR). Due to the inherent disadvantages of the customized fenestrated and/or branched aortic endografts (such as delayed cycles with a risk of aneurysm rupture, unavailable in emergency or confine operations), several off-the-shelf devices have been developed for the treatment of JRAAA. However, these devices being used in clinical trials have been proven to have a non-negligible risk of reintervention and inadequate anatomic applicability. We have developed a new off-the-shelf aortic endograft system (WeFlow-JAAA) with a mixed design of inner branches and modified fenestrations. The purpose of this cohort study is to assess the safety and effectiveness of the innovative aortic endograft system.

**Methods and analysis:**

This is a prospective, multicenter, single-armed clinical trial cohort study. The enrolment will take place in 29 centers in China, and 106 adult patients with JRAAA will be enrolled in total. Clinical information and CT angiography (CTA) images will be collected and recorded. Patients will be followed up for 5 years. The primary safety endpoint is the rate of no major adverse event within 30 days after index EVAR. The primary efficacy endpoint is the rate of immediate technical success and no JRAAA-related reintervention within 12 months after the procedure.

## Strengths and limitations of this study

• This is a prospective, multicentre, single-armed clinical trial cohort to evaluate the safety and efficacy of WeFlow-JAAA, a newly developed aortic endograft system, for the treatment of juxtarenal abdominal aortic aneurysms that are challenging for traditional endovascular aortic repair.
• A major strength of this study is that it evaluates a novel abdominal aortic endograft system based on a mixed design of inner branches and “mini-inner-cuff” reinforced fenestrations.
• The main limitations of this study are the regional limitation (China) and lack of control arms.

## Introduction

Juxtarenal abdominal aortic aneurysm (JRAAA) poses significant challenges in endovascular aortic repair (EVAR). In the European Society for Vascular Surgery (ESVS) 2019 clinical practice guidelines on the management of AAA, JRAAA is defined as an aneurysm extending up to but not involving the renal arteries, i.e., a short neck (<10 mm) (1). During the past 20 years, custom-made fenestrated and/or branched aortic endografts (i.e., Cook Zenith Fenestration) have largely replaced open surgery for the treatment of anatomically suitable JRAAA (2, 3), which is also recommended by the ESVS2019 clinical practice guidelines on the management of AAA (1).

Due to the inherent disadvantages of the customized device (delayed cycles with a risk of rupture, unavailable in emergency or confine operation) (4, 5), several “off-the-shelf” devices have been developed for the treatment of JRAAA, such as Cook p-Branch and Endologix Ventana (6, 7). According to a recent systematic review, clinical outcomes for the off-the-shelf devices in the treatment of complex AAA are promising, with no 30-day mortality, a 1.7% rate of paraplegia, a 97.6% 30-day target vessel patency rate, and a 3.3% rate of type I endoleak (8). However, high rates of late postoperative reintervention and narrow anatomic indications hampered the development of devices for JRAAA. Sveinsson et al. (7) found a 48% rate of reintervention during the 5 years follow-up of p-Branch. The device study of Endologix Ventana was also placed on hold for a high reintervention rate of renal arteries (9). Additionally, these off-the-shelf fenestrated devices are only anatomically suitable for 30–40% of all patients (10, 11). Thus, a safer and more effective off-the-shelf device with less reintervention and wider anatomic indications for the treatment of JRAAA is needed.

**FIGURE 1:**
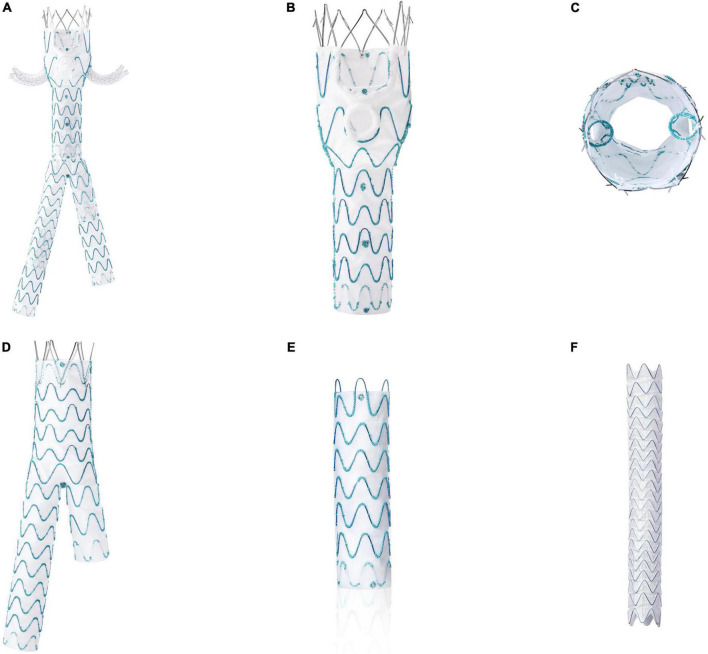
The WeFlow-JAAA system: a new, off-the-shelf, abdominal aortic stent graft system **(A)** for JRAAA, comprising a proximal body graft **(B,C)**, a distal bifurcated body graft **(D)**, iliac leg grafts **(E)** and self-expanding branch grafts **(F)**. The most major characteristic of this device was the design of proximal body graft with two standard inner branches of renal arteries, one 3 mm “mini-inner-cuff” reinforced fenestration for superior mesenteric artery, and one scallop for celiac axis.

In collaboration with Endonom Medtech (Hangzhou, China), we have developed a novel off-the-shelf modular endograft system (WeFlow-JAAA, Figure 1A) which consists of four components, a proximal body graft (Figures 1B,C), a distal bifurcated body graft (Figure 1D), iliac leg grafts (Figure 1E) and self-expanding branch grafts (Figure 1F). The proximal body graft (Figure 1C), with two standard inner branches (1.5 cm in length) of renal arteries (RA), one 3 mm “mini-inner-cuff” reinforced fenestrations with a sealing ring for superior mesenteric artery (SMA), and one scallop with a sealing ring for celiac axis (CA), has three important advantages. The first was inner branch design of bilateral renal arteries could significantly reduce branch instability or type III endoleak with matched branch stent grafts. The second advantage was that the modified fenestration reinforced with a 3 mm “mini-inner-cuff” and a sealing ring allows better seal at the attachment site, potentially reducing the risk of endoleak, without adding to the bulk of the device. The above two points lead to wider anatomic indications of this device, which was the third advantage. The anatomic indications of an aortic stent graft system depend on three aspects, including aortic related (especially the aortic diameter of the neck and branch level), branch vessel related, and approach related. The inner branch design could combine the benefits of external branch and fenestration and improve the anatomical fitness of aorta diameter. While the “mini-inner-cuff” reinforced fenestration could increase the sealing without compromising the approach-related anatomical fitness, which was precisely what the three preset guidewires (RAs, SMA) rather than preset catheters designed for.

The first-in-human (FIM) study (NCT04745546) of WeFlow-JAAA was initiated in October 2019 and is being conducted in a single center (data unpublished). This FIM study involved a total of 15 patients with 14 JRAAA and 1 suprarenal AAA, and all patients have currently completed at least 6 months of follow-up imaging. The main objective of this study was to evaluate the safety and effectiveness of this abdominal aortic stent graft system (WeFlow-JAAA) in preparation for its dissemination to maximumly benefit patients with JRAAA.

## Methods and analysis

This study was designed following the Standard Protocol Items “Recommendations for Interventional Trials statement” and is registered with the US National Institute of Health (Table 1). The protocol ID is WEIQIANG202101. The actual study start date was 23 February 2022, and the estimated study completion date is 31 December 2028.

**TABLE 1:** Trial registration data.

Date category	Information
Primary registry and trial identifying number	ClinicalTrials.gov NCT05179967
Date of registration in primary registry	6 January, 2022
Secondary identifying numbers	WEIQIANG202101
Source(s) of monetary or material support	Hangzhou Endonom Medtech Co., Ltd.
Primary sponsor	Hangzhou Endonom Medtech Co., Ltd.
Secondary sponsor(s)	None
Contact for public queries	Wei Guo, Professor, email: pla301dml@vip.sina.com
Contact for scientific queries	Wei Guo, Professor
Brief title	Safety and Efficacy Study of WeFlow-JAAA Stent Graft System for Complex Abdominal Aortic Aneurysm (GREAT Study)
Official title	Guo’s visceRal artEries Reconstruction: The Prospective, Multiple Center, Objective Performance Criteria Clinical Trial About the sAfTy and Efficacy of WeFlow-JAAATM Stent Graft System (GREAT Study)
Countries of recruitment	China
Problem(s) studied	Endovascular treatment for Juxtarenal Abdominal Aortic Aneurysm (JAAA)
Intervention(s)	Fenestrated/branched abdominal stent graft system
Key inclusion criteria	Age 18–80 years at the time of informed consent signature Maximum diameter of JRAAA > 50 mm, or rapid growth of sac > 5 mm in diameter in the most recent 6 months, or rapid growth > 10 mm in diameter with 1 year The distance between the upper edge of the aneurysm and the lower edge of the opening of SMA ≥ 4 mm The angle between the proximal aneurysm neck and the aortic long axis near the opening of SMA ≤ 60^°^ The aortic diameter at the opening of SMA ranges from 18 to 34 mm The diameter range of the starting part of SMA is 5–12 mm The diameter range of the initial part of bilateral RAs is 4.5–10 mm Length of non-bifurcated segment of SMA and RA ≥ 10 mm The diameter at the bifurcation of abdominal aorta ≥ 16 mm The length of distal anchoring area of iliac artery ≥ 15 mm The diameter range of distal anchoring area of iliac artery is 8–24 mm Feasible iliofemoral artery and upper patent upper extremity access
Key exclusion criteria	Ruptured aortic aneurysm in unstable hemodynamic instability Aneurysmal aortic dissection Infected or mycotic aortic aneurysm Local or systemic infection that may result in endoprosthesis infection Takayasu arteritis, Marfan syndrome (or other connective tissue diseases) Severe stenosis, calcification, and mural thrombosis in the proximal anchoring area of the stent Diagnosis of acute myocardial infarction within the last 3 months Transient ischemic attacks or strokes within the past 3 months A history of abdominal aortic surgery or endovascular repair Hepatic insufficiency comorbidity (ALT OR AST ≥ 5 times the upper limit of normal value, or total serum bilirubin ≥ 2 times the upper limit of normal value), serum creatinine ≥ 150 μmol/L, left ventricular ejection fraction < 50% Severe pulmonary insufficiency inability to tolerate general anesthesia Severe coagulation dysfunction An allergic history for anticoagulants, antiplatelet drugs, stent graft, or materials of the delivery system Contraindicated for antiplatelet agents or anticoagulants Serious vital organ dysfunction or other serious diseases Patients participating in other clinical trials or not completed or withdrawn from other clinical trials within 3 months at the time of the screening period Planning pregnancy, pregnancy, or breastfeeding Life expectancy < 1 year Patients not appropriate for endovascular repair based on the investigators’ clinical judgment
Study type	Interventional
Date of first enrolment	February 2022
Target sample size	106
Recruitment status	Recruiting
Primary outcome(s)	Safety outcome: rate of no major adverse event (time frame: 30 days after index endovascular procedure) Efficacy outcome: rate of immediate technical success and no JRAAA-related reintervention (time frame: within 12 months after index endovascular procedure)
Key secondary outcomes	Outcome: device-related complications (time frame: intraoperative and within 30 days after index procedure) Outcomes: Rate of all-cause mortality, aneurysm-related mortality (time frame: 30 days, 6, 12 months, and 2–5 years after index procedure)

JRAAA, Juxtarenal Abdominal Aortic Aneurysm; SMA, superior mesenteric artery; RA, renal artery; ALT, alanine transaminase; AST aspartate aminotransferase.

### Study design

This is a prospective, multicenter, single-arm, cohort study evaluating the safety and efficacy of a novel fenestrated and branched stent graft system for endovascular repair of JRAAA. This study is being initiated by the Chinese PLA General Hospital and will involve patients from an additional 28 high-volume tertiary referral hospitals across all geographic areas of China (Table 2). All participating hospitals have performed endovascular treatment in more than 50 patients with anatomically complex abdominal aortic aneurysms in the last 5 years.

**TABLE 2:** Trial centers.

Center	Geographic region
Chinese PLA General Hospital	North China
Beijing Anzhen Hospital, Capital Medical University	North China
Zhongshan Hospital, Fudan University	East China
The Second Xiangya Hospital of Central South University	Central China
People’s Hospital of Xinjiang Uygur Autonomous Region	Northwest China
The First Hospital of China Medical University	Northeast China
Peking University People’s Hospital	North China
West China Hospital of Sichuan University	West China
The First Affiliated Hospital, Sun Yat-sen University	South China
Nanjing Drum Tower Hospital	East China
The First Affiliated Hospital, Zhejiang University	East China
The Second Affiliated Hospital of Harbin Medical University	Northeast China
Fuwai Central China Cardiovascular Hospital	Central China
The First Affiliated Hospital of Fujian Medical University	South China
Jiangsu Province Hospital	East China
The First Affiliated Hospital of Zhengzhou University	Central China
The First Affiliated Hospital of Chongqing Medical University	Southwest China
Shandong Provincial Hospital	North China
The First People’s Hospital of Yunnan Province	Southwest China
Peking Union Medical College Hospital	North China
Shanghai Ninth People’s Hospital	East China
Tianjin Medical University General Hospital	North China
Xijing Hospital	Northwest China
The Second Affiliated Hospital of Nanchang University	East China
Qilu Hospital of Shandong University	North China
First Affiliated Hospital of Kunming Medical University	Southwest China
Yan’an Hospital of Kunming City	Southwest China
The First Affiliated Hospital of Harbin Medical University	Northeast China
The First Hospital of Lanzhou University	Northwest China

### Study endpoints

#### Primary safety endpoint

The primary safety endpoint is the rate of survival without major adverse events (MAEs) within 30 days after the index endovascular procedure. The MAEs include all-cause death, myocardial infarction, intestinal ischemia, renal failure needing continuous dialysis, respiratory failure, stroke, and permanent paraplegia.

#### Primary efficacy endpoint

The primary efficacy endpoint is the rate of clinical success within 12 months after the operation. Clinical success included immediate technical success (which is defined as correct system deployment without any unintentional occlusion of the aortic visceral branches or both hypogastric arteries, no type I or III endoleak, or conversion to open surgery), and no aneurysm or device-related death, no type I or III endoleak, no graft infection or thrombosis, no aneurysm expansion > 5 mm or rupture, no conversion to open surgery, no graft migration, or a failure of device integrity within 12 months after the procedure.

#### Secondary safety endpoints

The following secondary safety endpoints will be evaluated: all-cause mortality, aortic-related mortality, the incidence of device-related adverse events, and the incidence of serious adverse events (resulting in death or serious deterioration of health) before discharge and at 30 days, 6 months, 12 months, and 2–5 years postoperatively.

#### Secondary efficacy endpoints

The following secondary efficacy endpoints will be evaluated: the patency rate of postoperative branches, the incidence of type I/III endoleak, graft migration, open surgery conversion, and JRAAA-related reintervention at 6 months, 12 months, and 2–5 years postoperatively.

### Patient recruitments

**FIGURE 2:**
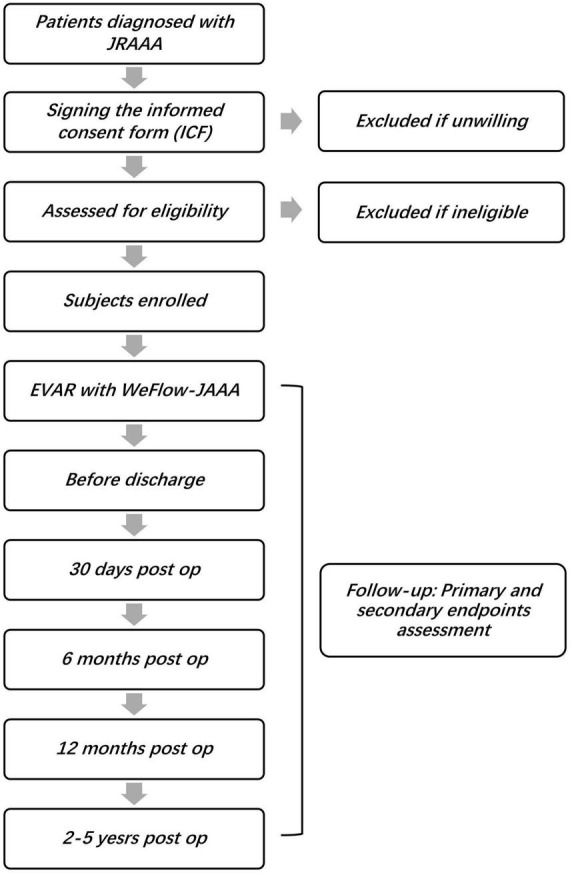
The study flow chart. (JRAAA, juxtarenal abdominal aortic aneurysm; EVAR, endovascular aortic repair; op, operation).

We are intent to recruit 106 patients between 18 and 80 years who are diagnosed with JRAAA. As the JRAAA is relatively common in clinical practice, the patient recruitment is non-competitive, and the enrollment number of each center shall be evenly distributed as far as possible to ensure the representativeness of the region. Of course, appropriate adjustments will be made according to the actual situation such as the enrollment process of each center. Figure 2 shows the time schedule of the study.

Table 1 gives a detailed overview of the inclusion and exclusion criteria. Vascular surgeons will judge the patients’ eligibility according to the criteria. Engineers from Hangzhou Endonom Medtech will provide on-site guidance regarding device manipulations for the early cases treated in participating hospitals.

### Device description

The design concept of WeFlow-JAAA is to revascularize the renovisceral arteries using a fenestration-and-branch mixed stent graft with two standard inner branches for the renal arteries, one 3 mm “mini-inner-cuff” reinforced fenestration for SMA, and one scallop for celiac axis (CA). The advantages of this mixed design have been proposed in the introduction.

The proximal body graft, constructed of woven polyester fabric sewn to self-expanding nitinol stents, consists of a bare stent part, an upper part (two fenestrations for SMA and CA, two inner branches for RAs), and a lower part that has a smaller diameter than the upper part (Figures 1B,C). A radiopaque “o” marker fixed at the 12 o’clock of the proximal end and two “I” markers are, respectively, fixed at the 4 and 8 o’clock positions of the proximal end (two sides of the scallop), coupled with one “o” marker between the scallop of CA and the fenestration of SMA to allow accurate positioning. The outlets and inlets of the inner branch and the fenestration of SMA are outlined by circular markers to facilitate cannulation. Two radiopaque “o” markers are positioned in the proximal end and distal end of the lower portion to identify the distal landing position and overlapping length with a distal bifurcated body graft.

The bare stent at the proximal end of the proximal body graft contains barbs for additional fixation of the device. The bare stent portion has lengths of 13 and 15 mm. The upper part of the proximal body graft has a diameter of 20–38 mm (2 mm increments) and lengths of 26 and 31 mm. The two inner branches sewn to the internal face of the upper part and the outlets are designed at the same level. The inner branches have three diameters available, 6, 7, and 8 mm, with a fixed length of 15 mm. The fenestration of SMA is positioned at 6 o’clock, with two diameters available, 8 and 10 mm. The lower part of the stent graft is 50–100 in length and 16–30 mm in diameter (2 mm increments). A total of 10 series with 41 types of WeFlow-JAAA endografts are available, all of which require a 22F inner diameter femoral sheath introducer, regardless of the stent graft size. To facilitate visceral artery cannulation, the delivery system of the system has three preloaded guidewires for the two inner branches and fenestration of SMA.

The self-expanding bridging branch stent graft (for the reno-visceral arteries) is constructed using a unique interwoven nitinol wire sandwiched by two thin layers of expanded polytetrafluorethylene (ePTFE), which can provide favorable flexibility, fracture resistance, and low-profile features. The branch graft diameter is 6–12 mm at the proximal end and 5–10 mm at the distal end, and the stent graft length is 20–150 mm. An inner 8–9F introducer sheath is required, depending on the stent graft diameter (8F: 6–9 mm; 9F: 10–12 mm).

The distal bifurcated body graft and iliac legs are constructed of woven polyester fabric sewn to self-expanding nitinol stents. The bifurcated body graft also has barbs in the proximal portion to prevent graft migration. A variety of sizes for distal bifurcated body graft and iliac legs are available to accommodate the different diameters of the proximal body graft and individual arterial anatomical variations.

The detailed product size series is shown in Supplementary Appendix 2.

### Endovascular technique

The index endovascular procedures are performed in a hybrid operating room under fluoroscopic control with the patient under general anesthesia. After surgical exposure of the left brachial artery, intravenous heparin loading is started with a bolus of 100IU/kg body weight to achieve an activated clotting time of at least 250 s. The activated clotting time would be measured at 30 min intervals throughout the procedure. A subsequent dose of heparin will be administered if required. The left brachial artery is directly punctured to establish the access through which the thoracic descending aorta is catheterized. An 8F long sheath is introduced into the descending thoracic aorta. The bilateral common femoral artery is percutaneously punctured and a 5/6Fsheath inserted. Two Perclose ProGlide devices were exchanged to provide “preclose” sutures on the main body side, while one on the contralateral side.

Detailed visualization of the operative procedure is shown in Supplementary Appendix 1. After the endovascular repair is finished, selective angiography is used to identify endoleaks, stent graft migration, stenosis, or occlusion. Relining bare metal stents are not routinely used unless a significant stent graft compression is noted.

### Medications

Single antiplatelet therapy (aspirin or clopidogrel) is recommended as a long-term treatment. Other medical treatments (such as anticoagulation, antihypertensive, antibiotic therapy, et al.) will be administered depending on the individual’s comorbidities.

### CT angiography

CT angiography (CTA) scans in the arterial and venous phases from the supra-aortic arch to the common femoral arteries will be performed preoperatively, before discharge, at 6 and 12 months postoperatively. CTAs are mandatory within 90 days and then at 1 and 5 years after the operation, or in case of unexpected events during the follow-up. For patients with substantially impaired renal function, postoperative CTA will be replaced by plain CT and Doppler ultrasonography.

### Patient timeline

Each patient will attend a total of 10 visits, including one preoperative visit and nine postoperative visits for eligibility screening, baseline data extraction, and safety/efficacy evaluation of the stent graft system (Table 3). Unless an unexpected event occurred, Doppler ultrasonography rather than CTA is planned for 2, 3, and 4 years postoperatively. Adverse events and defects of the devices will be strictly monitored throughout follow-up.

**TABLE 3:** Evaluation schedule of the study.

	Entry	Operation	Discharge	30 days (± 7 days)	6 months (± 30 days)	12 months (± 30 days)	24 months (± 60 days)	36 months (± 60 days)	48 months (± 60 days)	60 months (± 60 days)
Informed consent	X									
Eligibility screen	X									
Demographic data	X									
Medical/clinical history	X									
Vital signs	X	X	X							
Blood routine	X		X							
Urine routine	X		X							
Liver/renal function	X		X	X	X	X				
Coagulation function	X		X							
Enzymology test	X		X							
Pregnancy test	X									
Heart function	X		X							
Lung function	X									
CTA	X		X	X	X	X	*	*	*	X
DSA		X								
Operative recording		X								
Medications	X	X	X	X	X	X				
Adverse events	X	X	X	X	X	X	X	X	X	X

CTA, CT angiography; DSA, Digital Subtraction Angiography. *Duplex Ultrasound Scan (CTAs are mandatory within 90 days and then at 1 and 5 years after the operation, or in case of unexpected events during the follow-up).

### Data extraction, entry, and monitoring

The clinical database contains the data of participants’ electronic medical records (EMR), CTA images, and follow-up conditions. Preoperative data will be extracted from the EMR and entered into the database before the operation, while postoperative in-hospital data will be collected within 2 weeks after discharge. The headline of each patient’s DICOM format image at each timepoint will be modified before data extraction, with the identifying information (patient ID, name, date of birth) replaced by a random 6-digit number. Preoperative CTA image data will be extracted before the operation, while postoperative CTA image data will be extracted within 2 weeks after the image data is acquired. Postoperative CTA images obtained at each follow-up timepoint will be independently analyzed by two experienced vascular surgeons who are blinded to the CTA findings at the preoperative or other postoperative timepoints, patients’ EMR, and follow-up information. After the completion of data extraction, the discrete data from CTA scans will be integrated into one database according to the predesigned 6-digit number. All CTA images will be measured and assessed by the 3Mensio workstation (V.8.1; 3Mensio Medical Imaging). At least 10% of the total CTA scans (including preoperative and postoperative scans) will be randomly selected and re-analyzed by the two vascular surgeons to assess interobserver and interobserver agreement.

Data will be registered via an electronic data capture system (EDC) and centrally stored in a secure password-protected server. Double data entry, range checks, and logical consistency checking methods will be used to ensure the quality of data. Clinical research associates who are independent of the investigators will review the study documentation and records held at each site to ensure that the study is conducted in compliance with the regulatory requirements.

### Sample size calculation

Currently, no guiding principle exists regarding objective performance criteria (OPC) for a fenestrated/branched stent graft system in China. As the endpoints of this study differ from that of the p-Branch (off-the-shelf) and Zenith Fenestrations (customized) studies, the expected endpoint incidences were obtained based on data extracted from previous studies (7, 12). We hypothesized that the performance of the WeFlow-JAAA stent graft system will not be inferior to that of the p-Branch and be similar to that of Zenith fenestrations.

In this setting, combined with the current experience of clinical experts, it is estimated that the objective performance of clinical success rate at 12 months (the primary efficacy endpoint) is set to 70% and the target of WeFlow-JAAA is set to 85%. According to statistical requirements, we used 0.025 as α and 0.10 as β for a single-sided test. A sample size of 82 cases was thus obtained. Besides, the objective performance of the rate of survival without MAEs in 30 days (the primary safety endpoint) is set to 71% and the target of WeFlow-JAAA is set to 86%. As α = 0.025 and β = 0.10 for a single-sided test, a sample size of 80 patients is required. Both endpoints need to be achieved. Considering the shedding, the sample size increased by 20%. At least 103 subjects are required for this study eventually. Considering the facilitation of the study center allocation, it is expected to enroll 106 patients. The sample size is calculated as follows:


$$n={\frac{\left[Z_{1-\alpha /2}\,\sqrt{P_{0}\,\left(1-P_{0}\right)}+Z_{1-\beta }\,\sqrt{P_{T}(1-P_{T}}\right]}{{\left(P_{T}-P_{0}\right)}^{2}}}^{2}$$

(P_*T*_ presents the expected rate of the test instrument and P_0_ presents the target value).

### Statistical analysis

The analysis will be done on all participants of the study as a primary analysis population. Patient demographic data, safety endpoints, and efficacy endpoints will be analyzed descriptively, with continuous data expressed as means with SD or medians (IQR), and categorical data will be expressed as numbers and percentages.

The primary safety and efficacy endpoints will be summarized as numbers and percentages with their two-sided 95% exact CI, using the Clopper-Pearson method. In addition, the Kaplan-Meier method will be used to estimate the cumulative rate of each endpoint. Statistics calculations will be performed using SAS software V.9.4 (SAS Institute).

### Patient and public involvement

Patients or the public will not be involved in the design, conduct, reporting, or dissemination plans of our study.

## Ethics statement

The studies involving human participants were reviewed and approved by the Ethics Committee of Chinese PLA General Hospital. The patients/participants provided their written informed consent to participate in this study.

## Author contributions

J-PG and H-PZ drafted the manuscript. WG initiated the design of the study, while H-PZ, XJ, JX, X-HM, L-JW, M-HZ, and Y-LX helped with the implementation. All authors read and approved the final version of the manuscript.
