# Supplementary material for: A prospective, multicenter, single-arm clinical trial cohort to evaluate the safety and effectiveness of a novel stent graft system (WeFlow-JAAA) for the treatment of juxtarenal abdominal aortic aneurysm: A study protocol
Source: Front Cardiovasc Med. 2022 Sep 28;9:1013834. doi: 10.3389/fcvm.2022.1013834 (PMC9554137; doi:10.3389/fcvm.2022.1013834)
Supplement: Supplementary file 1 [file Data_Sheet_1.pdf]

Visualization of WeFlow-JAAA operation procedure

Vascular Department of Chinese PLA General Hospital

2022.8

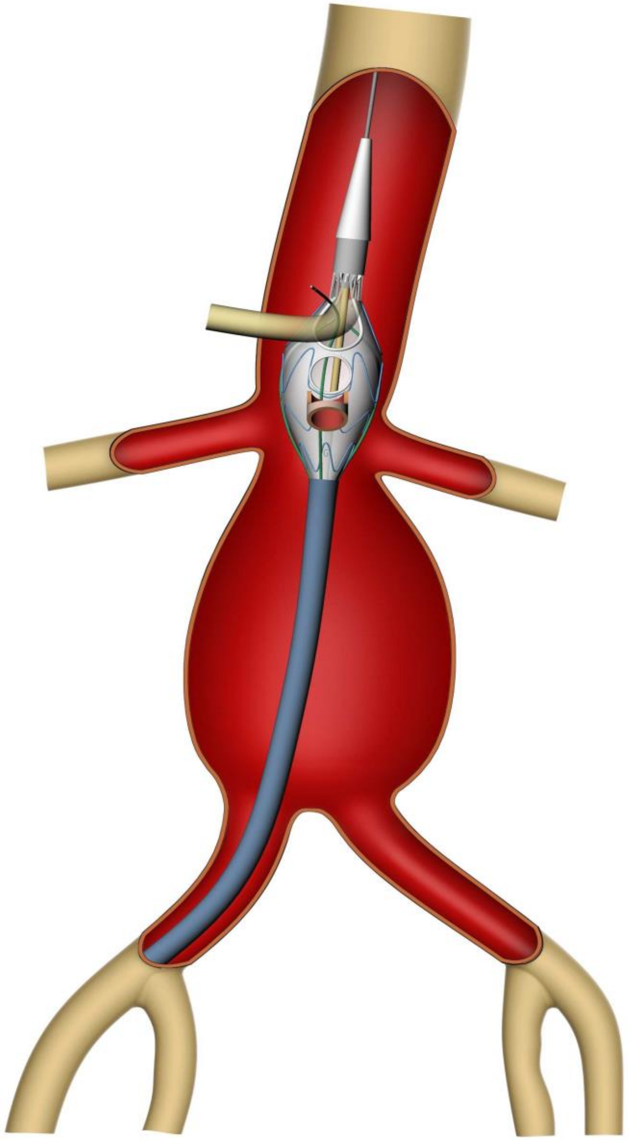

1. Advance the delivery system from the femoral access until the radiopaque markers indicating the modified fenestrations are at the appropriate level. Partially unsheathe the graft and ensure the anterior markers are in the most anterior position.

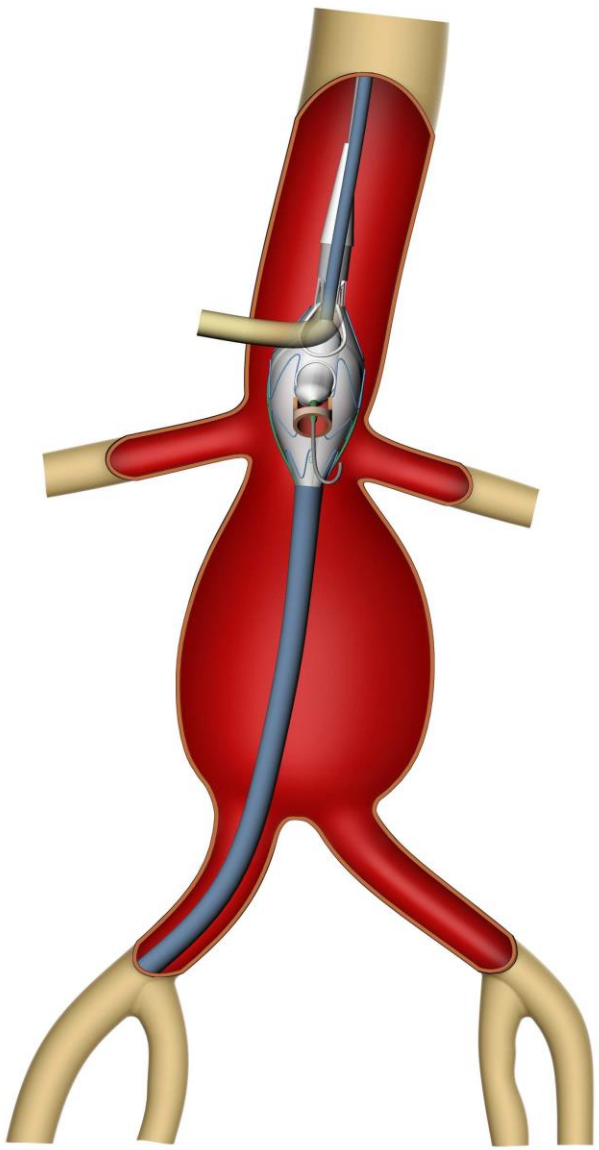

2. Use a gooseneck snare to captured the preloaded SMA guidewire and then catheterize the SMA from the established brachial-femoral access. Utilizing guidewire, an angioplasty balloon was advanced to the ostium of SMA and dilated to help stabilize the proximal body graft.

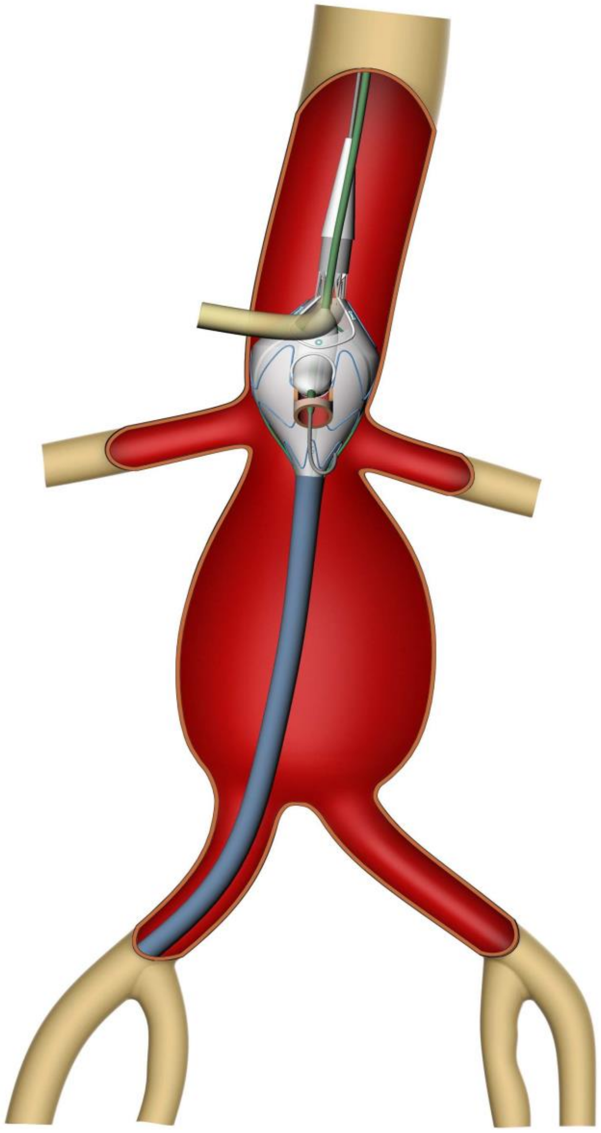

3. Maintain the femoral delivery system position and remove the diameter reducing tie of the proximal body.

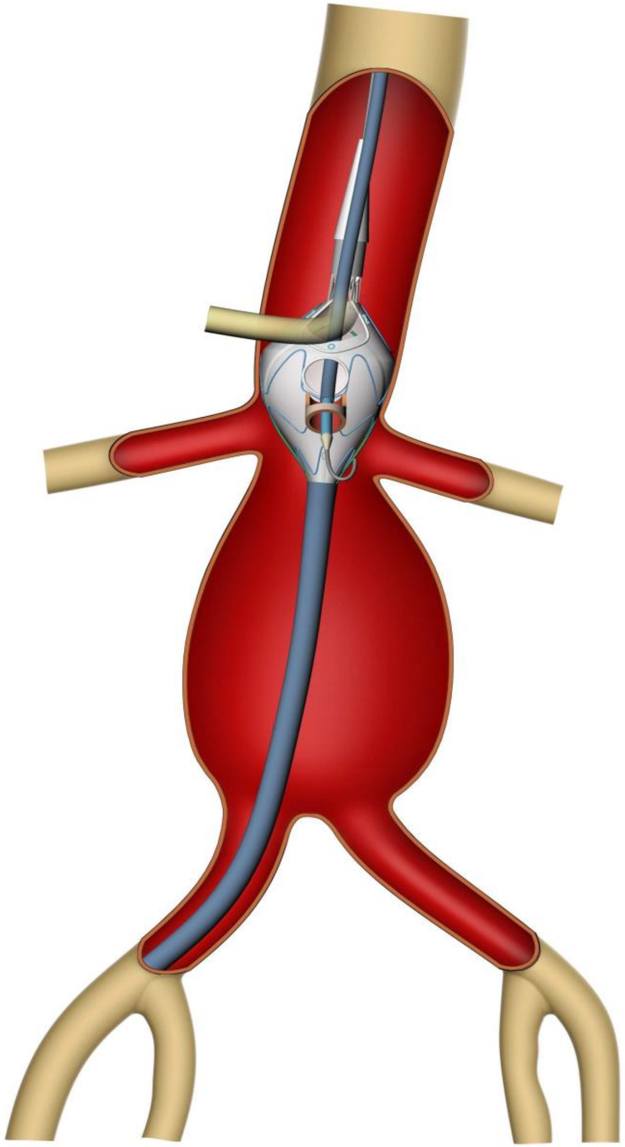

4. Introduce appropriately sized covered stent and advance partially into the SMA, leaving approximately 4-5mm of stent in the aorta.

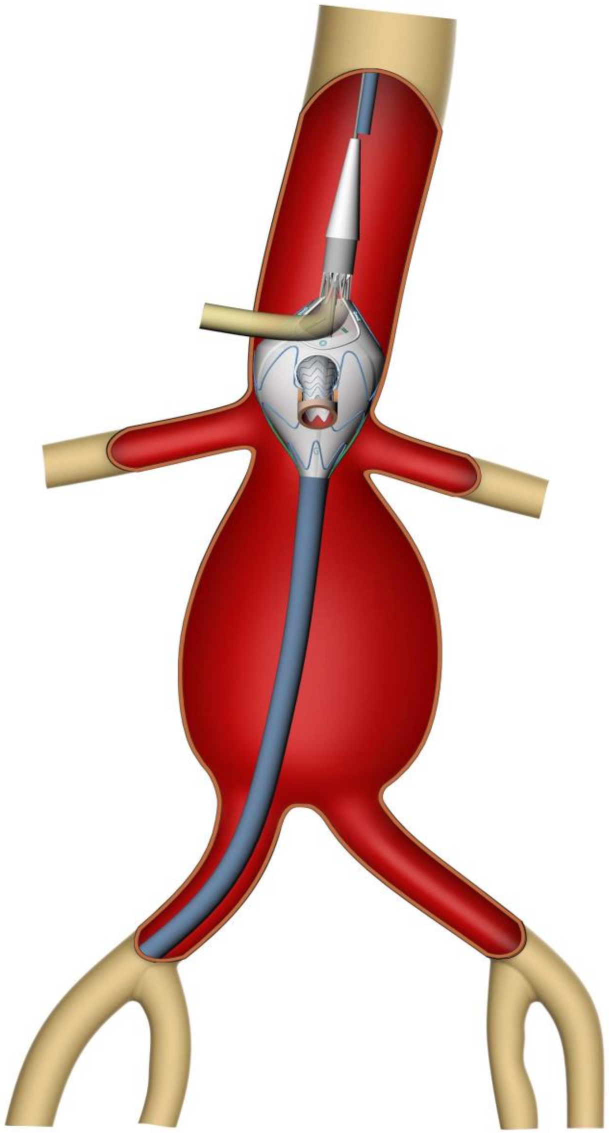

5. Release stent and flare the intra-aortic segment of the SMA stent with an angioplasty balloon to strengthen the seal.

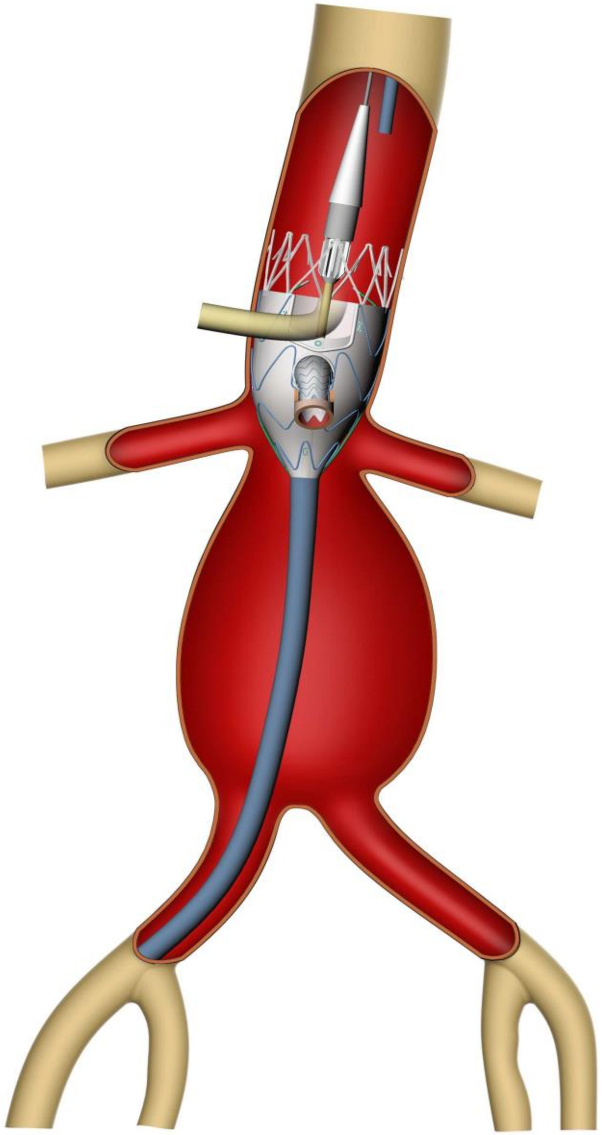

6. Remove the trigger-wire release mechanism to unlock the proximal stent from the top cap.

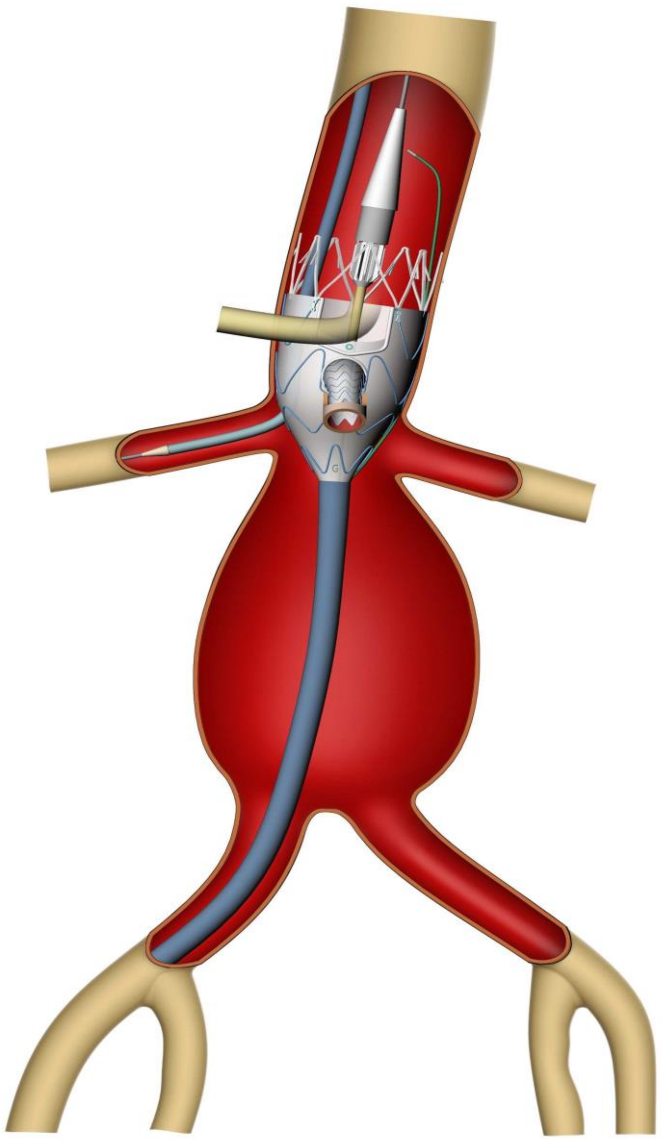

7. Establish the brachial-femoral access with the gooseneck snare and the preloaded right renal artery (RAA) guidewire. Catheterize the RRA and then advance an 8F long sheath into the inner branch with a balloon-supported passage technique. And then introduce an appropriately sized covered stent and advance into the RRA.

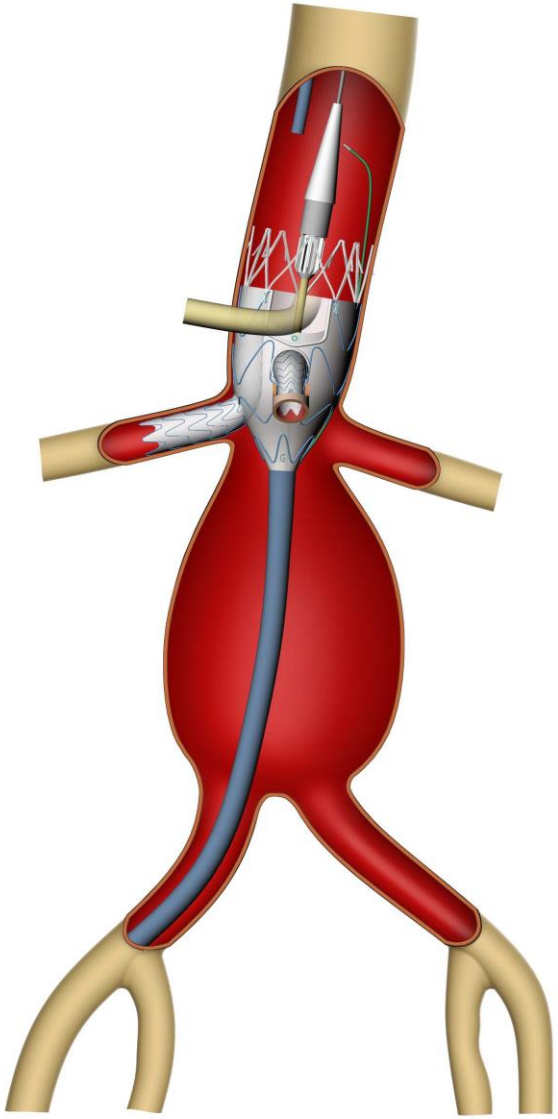

8. Release the covered stent and flare it with an angioplasty balloon to achieve a stable and smooth transition into the target vessel.

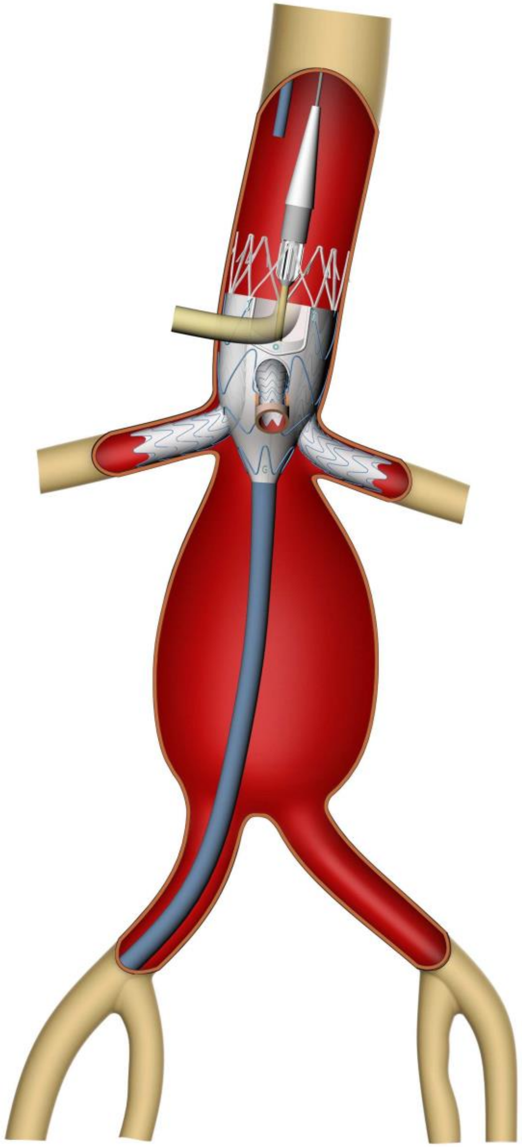

9. Revascularization of the left renal artery with the same method.

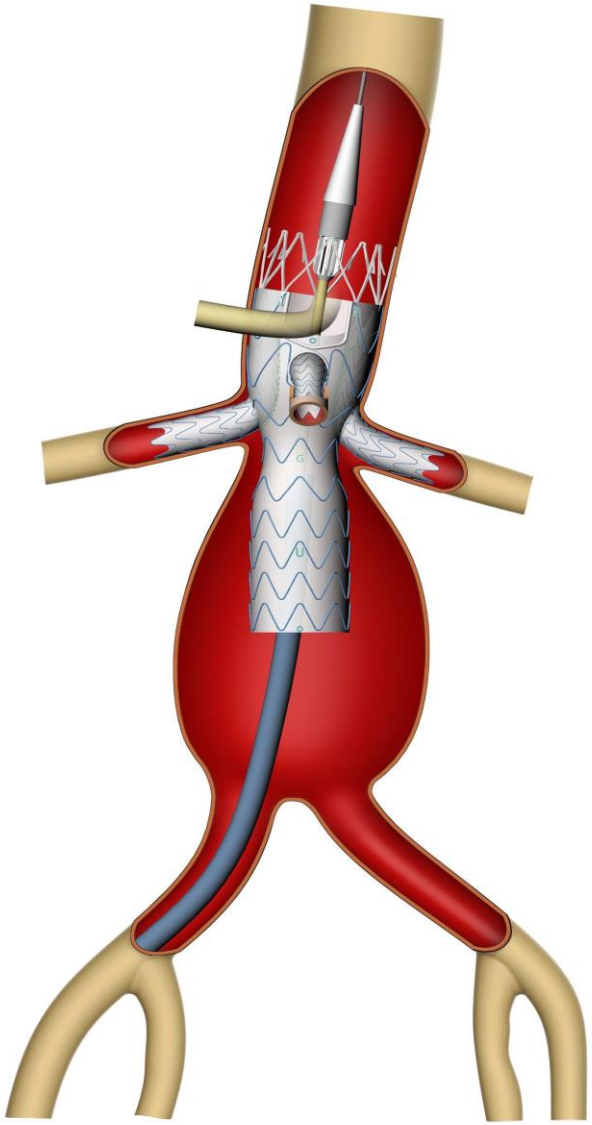

10. Stabilize the shaft of the deliver system while withdrawing the sheath until the proximal body graft has been fully unsheathed.

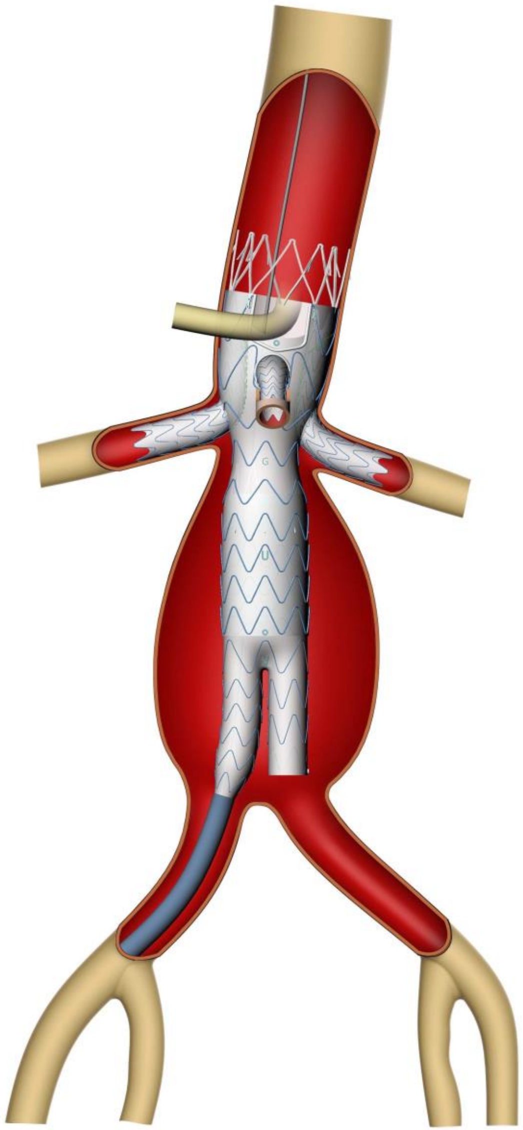

11. Insert and advance distal bifurcated body delivery system over the wire until the contralateral limb is positioned in suitable orientation above and anterior to the origin of the contralateral iliac. Withdraw the sheath until the contralateral limb is fully deployed.

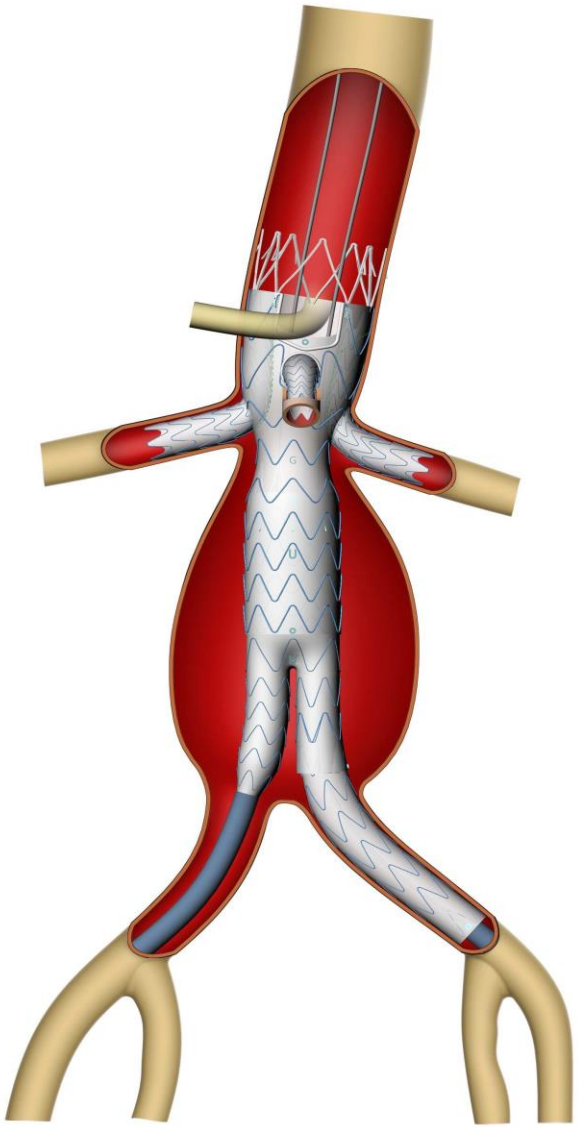

12. Manipulate the wire guide into the contralateral limb and the distal bifurcated body from the contralateral side. Perform angiography to confirm the proper position of the internal iliac artery, and then introduce and release the contralateral iliac leg graft.

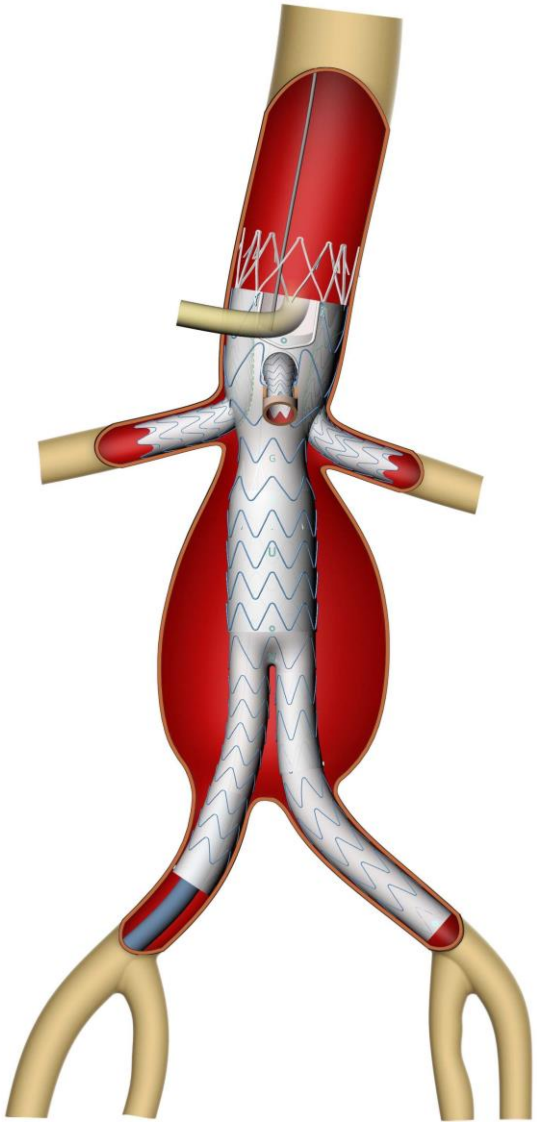

13. Withdraw sheath of the distal bifurcated body until the iliac leg is fully deployed. Place an additional iliac leg graft if necessary.

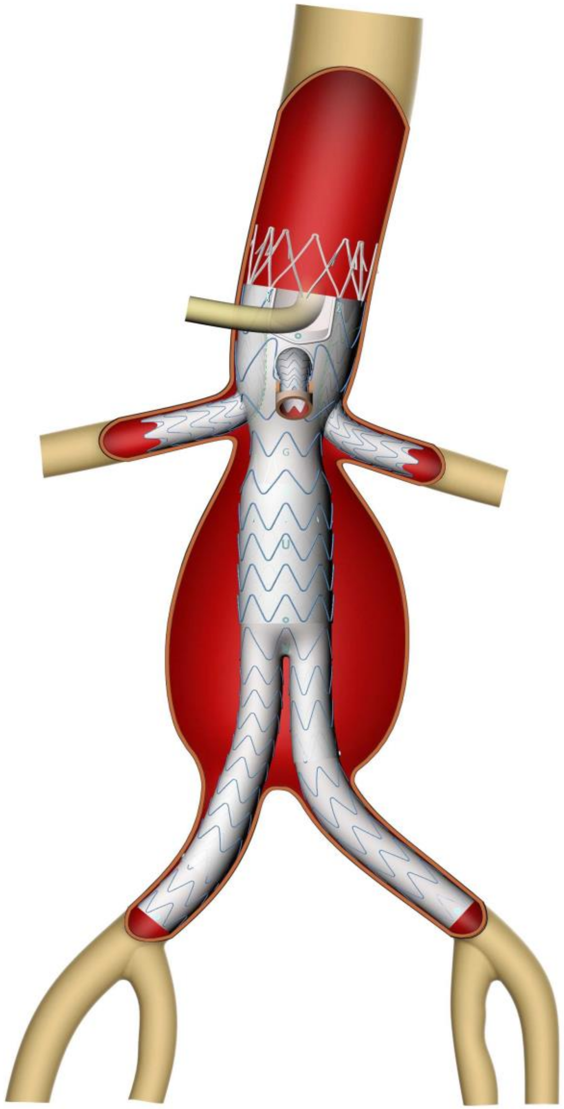

14. Mold the system with a molding balloon and finish the final angiogram.
