# Supplementary material for: A prospective, multicenter, single-arm clinical trial cohort to evaluate the safety and effectiveness of a novel stent graft system (WeFlow-JAAA) for the treatment of juxtarenal abdominal aortic aneurysm: A study protocol
Source: Front Cardiovasc Med. 2022 Sep 28;9:1013834. doi: 10.3389/fcvm.2022.1013834 (PMC9554137; doi:10.3389/fcvm.2022.1013834)
Supplement: Supplementary file 2 [file Data_Sheet_2.pdf]

TABLE I

Product size series of the proximal body graft (unit: mm)

<table>
<tr><th>Size series</th><th>Proximal diameter
D1±1.5</th><th>Distal diameter
D2±1.5</th><th>Length of Bare stent portion
L0±1</th><th>Length of the upper part
L1±2.5</th><th>Length of the lower part
L2±5</th><th>Branch diameter
D3±0.5</th><th>Fenestration diameter
D4±1</th><th>Size of the introducer sheath</th></tr>
<tr><td>20</td><td>20</td><td>16</td><td rowspan="4">13</td><td rowspan="10">26
31</td><td rowspan="10">50
60
70
80
90
100</td><td rowspan="10">6
7
8</td><td rowspan="10">8
10</td><td>22F</td></tr>
<tr><td>22</td><td>22</td><td>16/18</td><td>22F</td></tr>
<tr><td>24</td><td>24</td><td>16/18/20</td><td>22F</td></tr>
<tr><td>26</td><td>26</td><td>16/18/20</td><td>22F</td></tr>
<tr><td>28</td><td>28</td><td>16/18/20/22</td><td rowspan="6">15</td><td>22F</td></tr>
<tr><td>30</td><td>30</td><td>16/18/20/22/24</td><td>22F</td></tr>
<tr><td>32</td><td>32</td><td>16/18/20/22/24</td><td>24F</td></tr>
<tr><td>34</td><td>34</td><td>16/18/20/22/24/26</td><td>24F</td></tr>
<tr><td>36</td><td>36</td><td>18/20/22/24/26/28</td><td>24F</td></tr>
<tr><td>38</td><td>38</td><td>20/22/24/26/28/30</td><td>24F</td></tr>
</table>

Note: Figure I (next page)

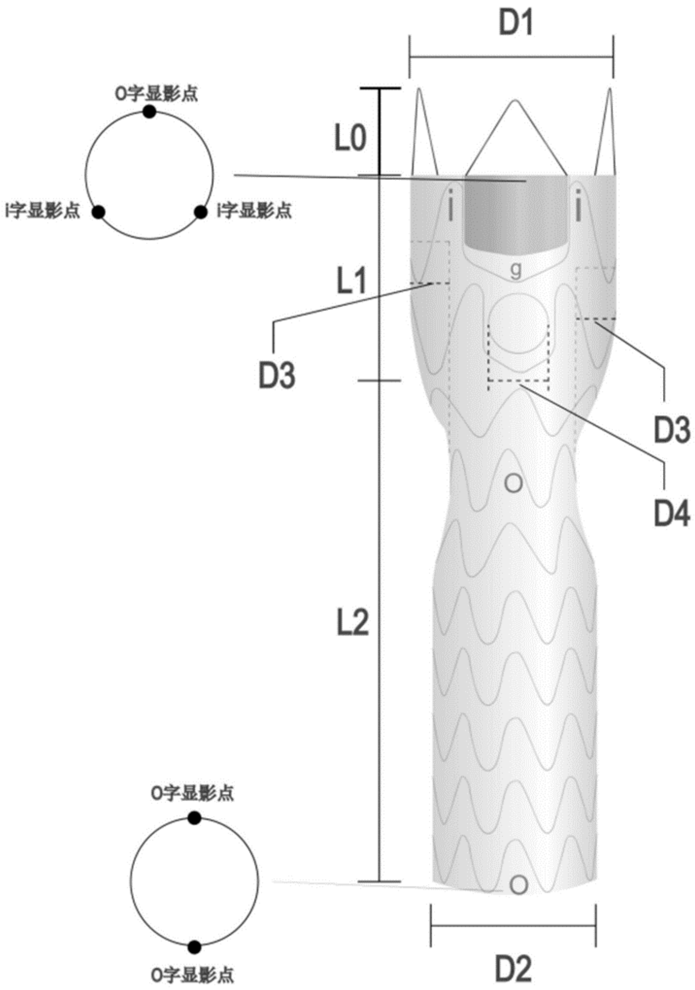

Figure I. Diagram of the proximal body graft

TABLE II
Product size series of the distal bifurcated body graft (unit: mm)

<table>
<tr><th>Size series</th><th>Diameter of the main body
D1±1.5</th><th>Diameter of the iliac branch
D2±1.5</th><th>Length of Bare stent portion
L0±1</th><th>Length of the main body
L1±5</th><th>Length of the long branch
L2±5</th><th>Length of the short branch
L3±3</th><th>Size of the introducer sheath</th></tr>
<tr><td>18</td><td>18</td><td>09/10</td><td rowspan="10">7</td><td rowspan="10">40
50
60
70
80</td><td rowspan="10">60
70
80
90
100</td><td rowspan="10">30</td><td>18F</td></tr>
<tr><td>20</td><td>20</td><td>12/14</td><td>18F</td></tr>
<tr><td>22</td><td>22</td><td>12/14</td><td>18F</td></tr>
<tr><td>24</td><td>24</td><td>14</td><td>18F</td></tr>
<tr><td>26</td><td>26</td><td>14</td><td>18F</td></tr>
<tr><td>28</td><td>28</td><td>14</td><td>18F</td></tr>
<tr><td>30</td><td>30</td><td>14</td><td>18F</td></tr>
<tr><td>32</td><td>32</td><td>14</td><td>18F</td></tr>
<tr><td>34</td><td>34</td><td>14</td><td>18F</td></tr>
<tr><td>36</td><td>36</td><td>14</td><td>18F</td></tr>
</table>

Note: Figure II (next page)

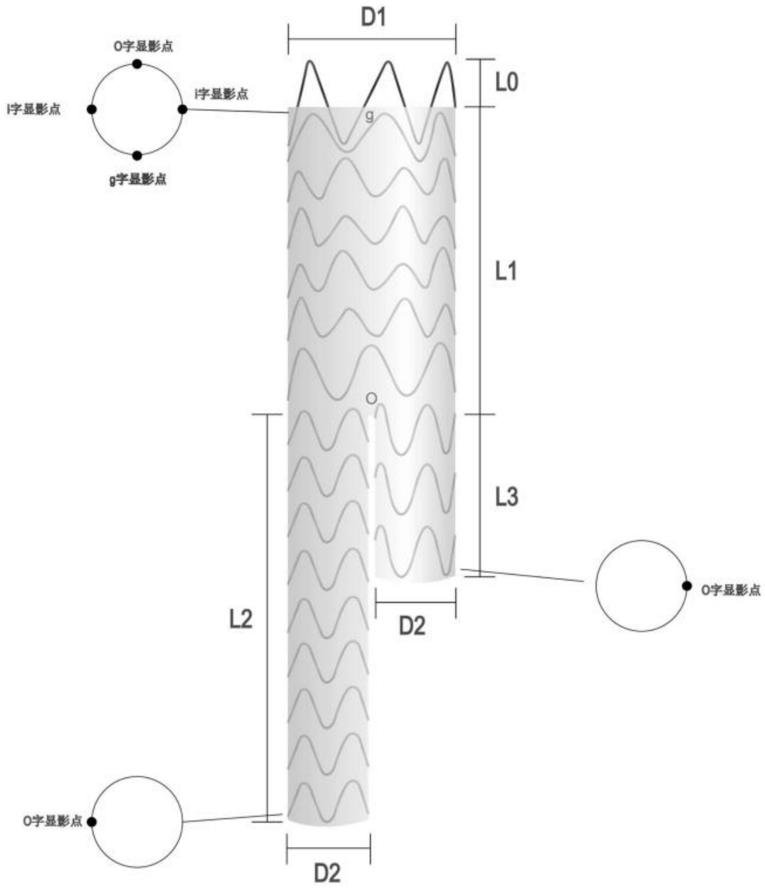

Figure II. Diagram of the distal bifurcated body graft

TABLE III

Product size series of the iliac branch graft (unit: mm)

Size series	Distal diameter<br>D1±1.5	Proximal diameter<br>D2±1.5	Length<br>L1±5	Size of the introducer sheath
10	10	10<br>12<br>14<br>16	50/60/70/80/<br>90/100/120/<br>140/160	16F
12	12			16F
14	14			16F
16	16			16F
18	18			16F
20	20			16F
22	22			16F
24	24			16F
26	26			16F
28	28			16F

Note: Figure III (right side)

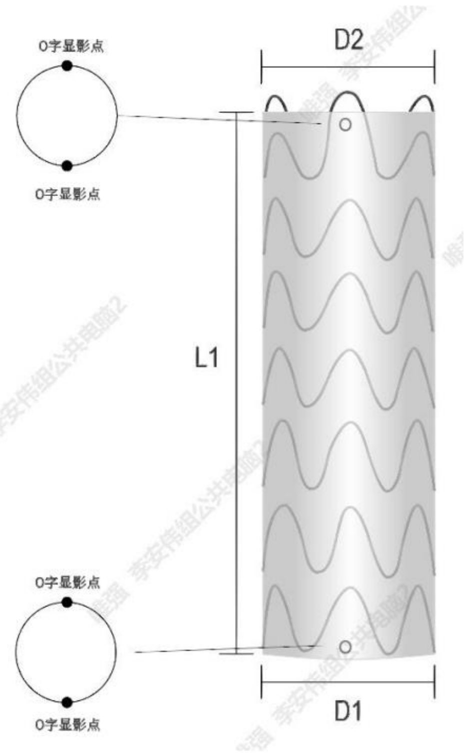

Figure III. Diagram of the iliac branch graft

TABLE IV

Product size series of the bridging branch graft (unit: mm)

Size series	Distal diameter (branch side)	Proximal diameter (Main body side)	Length of the branch	Size of the introducer sheath
	D1±1.0	D2+1.0/-0.5	L1±5	
5/6/7/8/9	5/6/7/8/9	6/7/8/9	20/30/40/50/60/70/80/90/100	8F
			120/140/150	9F
		10/11/12	20/30/40/50	8F
			60/70/80/90/100/120/140/150	9F
10/11	10/11	6/7/8/9/10/11/12	20/30/40/50/60/70/80/90/100	8F
			120/140/150	9F
12/13/14	12/13/14	6/7/8/9/10/11/12	20/30/40/50/60/70/80/90/100/120/140/150	9F
				9F

Note: Figure IV (next page)

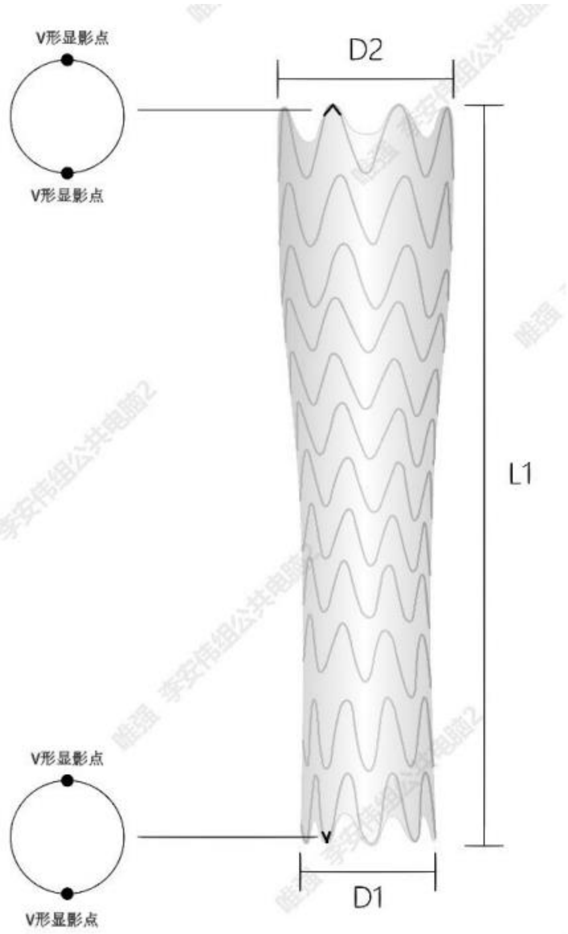

Figure IV. Diagram of the bridging branch graft
